# Pulse oximetry training landscape for healthcare workers in low- and middle-income countries: A scoping review

**DOI:** 10.7189/jogh.13.04074

**Published:** 2023-09-22

**Authors:** Meagan E Peterson, Shgufta Docter, Daniel R Ruiz-Betancourt, Jude Alawa, Sedera Arimino, Thomas G Weiser

**Affiliations:** 1 Stanford University School of Medicine, Stanford, California, USA; 2 School of Medicine, University of Limerick, Limerick, Ireland; 3 CHRR (Regional Hospital Centre of Reference) Vakinankaratra, Madagascar; 4 Department of Surgery, Stanford University, Stanford, California, USA

## Abstract

**Background:**

Pulse oximetry has been used in medical care for decades. Its use quickly became standard of care in high resource settings, with delayed widespread availability and use in lower resource settings. Pulse oximetry training initiatives have been ongoing for years, but a map of the literature describing such initiatives among health care workers in low- and middle-income countries (LMICs) has not previously been conducted. Additionally, the coronavirus disease 2019 (COVID-19) pandemic further highlighted the inequitable distribution of pulse oximetry use and training. We aimed to characterise the landscape of pulse oximetry training for health care workers in LMICs prior to the COVID-19 pandemic as described in the literature.

**Methods:**

We systematically searched six databases to identify studies reporting pulse oximetry training among health care workers, broadly defined, in LMICs prior to the COVID-19 pandemic. Two reviewers independently assessed titles and abstracts and relevant full texts for eligibility. Data were charted by one author and reviewed for accuracy by a second. We synthesised the results using a narrative synthesis.

**Results:**

A total of 7423 studies were identified and 182 screened in full. A total of 55 training initiatives in 42 countries met inclusion criteria, as described in 66 studies since some included studies reported on different aspects of the same training initiative. Five overarching reasons for conducting pulse oximetry training were identified: 1) anaesthesia and perioperative care, 2) respiratory support programme expansion, 3) perinatal assessment and monitoring, 4) assessment and monitoring of children and 5) assessment and monitoring of adults. Educational programmes varied in their purpose with respect to the types of patients being targeted, the health care workers being instructed, and the depth of pulse oximetry specific training.

**Conclusions:**

Pulse oximetry training initiatives have been ongoing for decades for a variety of purposes, utilising a multitude of approaches to equip health care workers with tools to improve patient care. It is important that these initiatives continue as pulse oximetry availability and knowledge gaps remain. Neither pulse oximetry provision nor training alone is enough to bolster patient care, but sustainable solutions for both must be considered to meet the needs of both health care workers and patients.

Pulse oximetry has been used in medical care since the 1970s; a decade later its use became standard of care in high resource settings, first in the perioperative space and subsequently for use in routine vital sign monitoring [1-3]. Numerous multilateral organisations and global public health initiatives associated with them recommend increased pulse oximetry training and use, from surgical and anaesthesia initiatives to child health and welfare programmes [4-7]. Despite this, pulse oximetry is not universally available, and the COVID-19 pandemic has further highlighted the inequitable distribution of pulse oximetry use and training, among other key health care capacity measures [8,9].

In the perioperative setting, pulse oximetry use in low-resource settings has lagged behind use in high-resource settings due, in part, to decreased oximetry availability and non-universal training initiatives for all health care workers [10]. Studies have demonstrated a need for continued strengthening of pulse oximetry use and training given identified capacity and knowledge gaps across multiple countries and additional practice areas, including trauma, obstetrics, paediatrics and neonatology [11-16].

Pulse oximetry training initiatives have been ongoing for years, but a map of the literature describing such initiatives among health care workers in low- and middle-income countries (LMICs) has not previously been conducted. To continue making progress in strengthening health care delivery, it is important to understand what pulse oximetry training initiatives have been done, the settings in which they have been conducted, and the health care workers who have been prioritised for training. We aimed to characterise the landscape of pulse oximetry training for health care workers in LMICs prior to the COVID-19 pandemic as described in the literature. To do this, we aim to address the following evaluation questions as part of our scoping review: 1) who (i.e. what type of health care workers) are being trained to use pulse oximetry in LMICs?; 2) what resources are used and how is training structured?; 3) when have they been trained?; 4) where are they being trained?; 5) why are they being trained (i.e. for what application of pulse oximetry)?; 6) how effective has the training been?

## METHODS

### Protocol

We developed a protocol *a priori* using the Preferred Reporting Items for Systematic Reviews and Meta-analysis Protocols (PRISMA-P). Given the aim to map literature related to pulse oximetry training among health care workers in LMICs prior to the COVID-19 pandemic, a scoping review was deemed the most appropriate methodology [17]. This scoping review is reported according to Preferred Reporting Items for Systematic Reviews and Meta-Analyses extension for Scoping Reviews (PRISMA-ScR) checklist presented in Table S1 in the **Online Supplementary Document** [18].

### Search strategy and sources of evidence

We systematically searched 6 databases (PubMed, Embase, CINAHL, Cochrane Library, Scopus, and World Health Organization (WHO) Global Index Medicus) to identify studies reporting pulse oximetry training among health care workers, broadly defined, in LMICs prior to the COVID-19 pandemic. Search terms and MeSH headings related to pulse oximetry, LMIC countries, and education initiatives were developed in PubMed and adapted for other databases in collaboration with a specialist librarian presented in Table S2 in the **Online Supplementary Document**. We searched databases from their inception to 14 May 2021 and made no language exclusions. No exclusions were made based on study design and letters to the editor and editorials were also assessed for inclusion criteria if they were identified in the database searches. References of identified studies and relevant reviews and editorials were also reviewed. We attempted to locate full texts of studies for relevant conference abstracts and study protocols through hand searching. If no full study could be identified, protocols were excluded and abstracts were included if there was enough information for data charting; otherwise, they were also excluded.

### Assessment of eligibility

We imported all references to Covidence (Covidence systematic review software, Veritas Health Innovation, Melbourne, Australia. Available at www.covidence.org), where duplicates were removed. Two reviewers (MEP and SD or DRRB) independently assessed titles and abstracts and relevant full texts for eligibility. Studies were eligible if they 1) described training health care workers from an LMIC as defined by The World Bank in calendar year 2020 [19], 2) described educational interventions surrounding the use of pulse oximeters and 3) the educational initiative occurred prior to 2020. Of note, pulse oximetry did not need to be the primary focus of the educational initiative. Studies were excluded if they 1) did not conduct pulse oximetry training in an LMIC, 2) only described the introduction of pulse oximeters without mention of any training received, such as capacity or knowledge assessments without an education or training component, or 3) pulse oximetry training was not conducted for health care workers.

### Evidence synthesis

Data were charted using forms created in Covidence by one author (MEP, DRRB, JA or SA) and reviewed for accuracy by a second (MEP, DRRB, JA or SA), with a singular author (MEP) completing one of these steps for all included studies. Discrepancies were discussed between the two authors and a final decision made. Items charted from each study included: 1) training setting, 2) year of training, 3) population being trained, 4) reason for training, 5) training structure (pulse oximetry training materials used and length of training, when reported) and 6) pulse oximetry specific outcomes of training, when reported. If pulse oximetry specific outcomes were not reported, an alternative relevant study outcome was charted. We synthesised the results using a narrative synthesis.

## RESULTS

**Figure 1:**
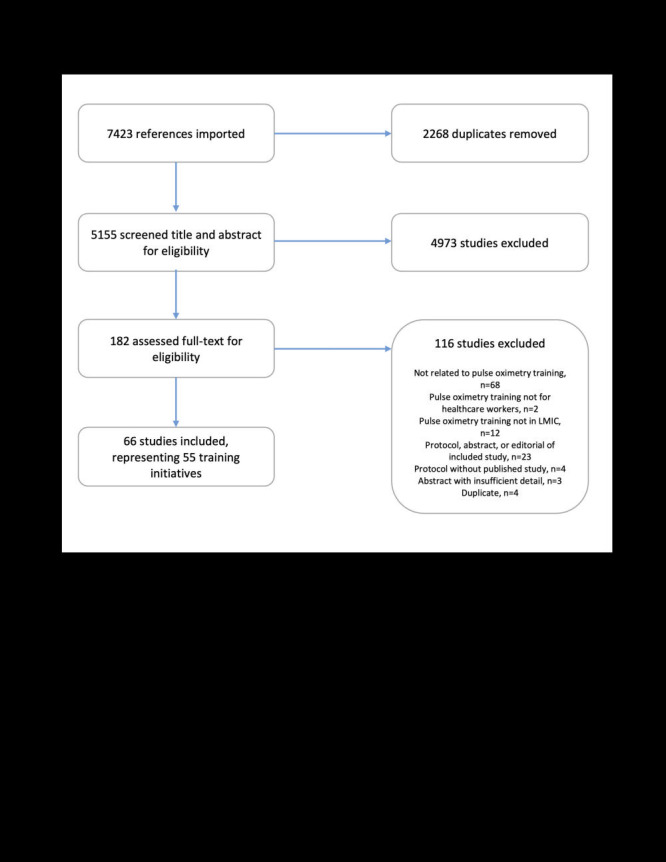
PRISMA flow diagram.

A total of 7423 studies were identified and 182 screened in full. Of these, 66 met our inclusion criteria (**Figure 1**). A total of 55 training initiatives were identified in 42 countries, with some included studies reporting on different aspects of the same training initiative. Five overarching reasons for conducting pulse oximetry training were identified: 1) anaesthesia and perioperative care, 2) respiratory support programme expansion, 3) perinatal assessment and monitoring, 4) assessment and monitoring of children and 5) assessment and monitoring of adults.

Training initiatives utilised a variety of structures, teaching styles and methods of assessment. They ranged from solely focused on pulse oximetry to being broader in their focus with pulse oximetry training being a small component of the overall educational agenda. Similarly, training ranged from a brief one-hour lecture to multiday workshops with practical skills assessments participants were required to pass. Some initiatives were longitudinal in nature and followed up with trainees overtime to assess continued pulse oximetry competency and use, while others studies’ only touchpoint with trainees was at the time of the initial training session. Such differences are further described in detail below in each respective section and results table.

### Anaesthesia and perioperative care

Twenty-four studies described conducting pulse oximetry training focused on perioperative teams in 21 countries globally, including 15 in the African Region, one in the European Region, three in the South-East Asia Region, and two in the Western Pacific Region (**Table 1**). Of these, nine had low-Income designation, nine lower-middle income and three upper-middle income. The primary foci of these efforts involved anaesthesia capacity-building and establishing the use of the World Health Organization Surgical Safety Checklist (WHO SSC). Training focused on anaesthesia providers, surgeons and nurses. Although training structure, length, and evaluation methods differed, many studies reported an increased use of pulse oximetry after training. Often, pulse oximetry capabilities were limited by availability of pulse oximeters and, secondarily, staff trained in its use. Multiple studies report increased intraoperative monitoring as well as the establishment of postoperative monitoring capabilities after training and pulse oximetry provision.

**Table 1:** Studies describing pulse oximetry training initiatives: Anaesthesia and perioperative care*

WHERE: country (LMIC designation), sites	WHEN: year of training	WHO: population trained	WHY: purpose of training	WHAT: structure of training	HOW: pulse oximetry related outcomes
**African Region**					
Benin (UMIC), 36 hospitals nationwide [20]	2016-2017	Surgeons, anaesthesia providers, nurses, and other perioperative staff	SSC implementation	3-d Mercy Ships led workshop	Always using a pulse oximeter increased from 86.6 to 97.0% 12-18 mo after training among 17 hospitals selected for follow-up.
Burkina Faso (LIC), 57 hospitals nationwide [21]	2013	Anaesthetists	To improve the practice of pulse oximetry and SCC implementation	Lifebox workshop	Systematic use of pulse oximetry during anaesthesia increased from 73% to 100% of hospitals. Prior to training, 17% of hospitals had a PACU and used the pulse oximeter from theatre to monitor patients post-operatively. After training, 94% of hospitals used post-operative pulse oximetry monitoring.
Cameroon (LMIC), 25 hospitals nationwide [22]	2017-2018	Operating room staff	SSC implementation	3-d multidisciplinary training course developed by Mercy Ships	“Always” or “often” intraoperative pulse oximetry use increased from 74% before training to 93% 4 mo after training.
Republic of Congo (LMIC), 1 hospital in Dolisie [23]	2014	Operating team personnel	SSC implementation	4-d pilot SSC training course developed by Mercy Ships	Intraoperative pulse oximetry “always” use increased from 0% before training to 86% 15 mo after training.
Ethiopia (LIC), 1 hospital in Addis Ababa [24]	2011-2012	Plastic and reconstructive surgery surgeons, anaesthetists, nurses, and other perioperative staff	Implement anaesthetic pre-assessment, SSC, continuous pulse oximetry monitoring in recovery areas, improved observation protocols in recovery areas, and the development of an HDU	Teaching sessions and simulation workshops	Pulse oximetry used intraoperatively in 98% of cases during the 8 mo after training. Continuous pulse oximetry available for all recovery beds after training.
Ethiopia (LIC), 1 hospital in Addis Ababa (primarily), 9 hospitals in southwestern Ethiopia (pulse oximeters only) [25]	2012-2018	Anaesthesia providers	Improve morale and retention, establish postgraduate physician training, SSC implementation, develop PACU	Lifebox workshop	Pulse oximeters: 6-mo follow-up showed retained pulse oximetry knowledge and use. SSC: >90% use of SSC. PACU: Patients now monitored postoperatively in PACU.
Ghana (LMIC), 1 nurse anaesthetist school in Kumasi [26]	Since 1987	Nurse anaesthetists	To improve anaesthetic patient care and safety	18-mo training programme in collaboration with University of Utah	In 2000, pulse oximetry was not used in the affiliate hospital. In 2009, 70% of the district and regional hospitals use pulse oximetry, including in PACUs.
Ghana (LMIC), 1 nurse anaesthetist school in Accra [27]	Since 2009	Nurse anaesthetists	To increase the number of anaesthesia providers	18-mo training programme	95% of graduates (representing 39 hospitals across 7 of the 10 regions) surveyed had access to pulse oximetry at their hospital.
Guinea (LIC), 6 hospitals nationwide [28]	2012-2013	Surgeons, anaesthetists, and nurses	To evaluate three different methods of SSC implementation	Training delivered by Mercy Ships: 1) team training in operating room and classroom (surgeon AND anaesthesia provider or nurse); 2) individual training in operating room and classroom (surgeon or anaesthesia provider); 3) individual training in the classroom only (anaesthesia provider)	4 of 6 hospitals had no pulse oximetry. Pulse oximetry was occasionally available in the other 2. No pulse oximeters were provided as part of the study. However, participants agreed that it would be a valuable addition in the OR and recovery wards. Multidisciplinary courses more impactful than single discipline at 3-6 mo.
Kenya (LMIC), 3 sub-district hospitals in Western Kenya [29]	2013-2014	Non-anaesthetist clinicians (nurses, clinical officers, medical officers, nurse aid)	Safer surgical care when no anaesthetist is available	5-d Every Second Matters-Ketamine (ESM-Ketamine) training course	Surgeries able to be performed when ESM-Ketamine protocol enacted, pulse oximeters alerted desaturation events.
Liberia (LIC), 2 hospitals in Monrovia [30]	2008-2009	Surgical team	SSC implementation	2-week training programme	Pulse oximetry use increased from 34.9% to 88.2% in Hospital 1 and 75.7% to 88.5% in Hospital 2 after training.
Madagascar (LIC), 21 hospitals nationwide [31-33]	2015-2016	Operating room staff	SSC implementation	3-d multidisciplinary course	Prior to training, no hospital routinely used pulse oximetry due to lack of supply. 3-4 mo after training, 63% of participants surveyed reported “Always” using pulse oximetry in theatre and 11% using it “most of the time.” 12-18 mo after training, 88% of participants surveyed reported “Always” using pulse oximetry in theatre and 9% using it “most of the time.”
Malawi (LIC), 27 hospitals [34]	2014	Anaesthesia providers	Perioperative monitoring	1-d Lifebox workshop	Improved pulse oximetry knowledge via MCQs immediately after training that was maintained after 8 mo. 82% of donated pulse oximeters were located at follow up. 97% of located pulse oximeters were in regular use at follow up. 8% relative reduction in the odds of desaturation event for every 10 cases during first 100 cases after training.
Niger (LIC), 40 public hospitals nationwide [35]	2014	Anaesthesiologists and surgeons	Pulse oximetry use, hypoxia management, implementation of SSC	Lifebox workshop	Average number of pulse oximeters in each hospital increased from 1 to 8. Logbook for notification and management of hypoxia introduced.
Togo (LIC), providers nationwide [36]	2012	Anaesthesia providers	To improve surgical and anaesthesia safety	Lifebox workshop	An audit of a maternity unit in 2014 demonstrated all patients receiving anaesthesia were monitored with pulse oximetry perioperatively and pulse oximetry training and provision enabled early hypoxia detection and interventions.
Uganda (LIC): 12 hospitals nationwide [37]	2007	Anaesthesia providers	Identify pulse oximetry gaps and training needs for perioperative monitoring	2 half-day Global Oximetry project workshop with refresher 1 y later	Test scores improved for all but two participants after training. All participants were able to demonstrate basic oximetry use after training. Demonstrating a change of practice.
Uganda (LIC), providers nationwide [38]	2011	Non-physician anaesthetists	Oximetry and hypoxia management	2.5 d Lifebox training course	Pulse oximetry knowledge improved from a median score of 36 / 50 to 41 / 50 (*P* < 0.0001) immediately after course. 3-5 mo later, the median score was 41 / 50 (*P* = 0.001 compared with immediate post-training test scores), and 95% of oximeters were in routine clinical use. Participants felt oximeters improved patient safety.
Zambia (LMIC), no site specified [39]	No year noted. Abstract presented in 2016.	Physicians and clinical officers throughout Zambia	Improve anaesthesia capacity, SSC, and pulse oximetry monitoring to reduce maternal mortality	1-d Lifebox workshop and 3-d Safe Anesthesia From Education (SAFE) obstetric anaesthesia courses	Lifebox MCQ, SAFE MCQ, and SAFE skills scores all improved after the training course.
**European Region**					
Moldova (UMIC), 1 hospital in Chisinau [40]	2010	Operating Room Staff	SSC implementation	Train-the-trainer approach with months long progressive rollout using course materials developed by WHO, Harvard School of Public Health, the World Federation of Societies of Anaesthesiologists, and the Association of Anaesthetists of Great Britain and Ireland, and intraoperative teaching	Pulse oximeters in operating stations increased from 14 to 100%. Pulse oximetry use in cases increased from 16 to 99.6%. Hypoxemic episodes lasting 2 min or longer per 100 h of oximetry decreased from 11.5 to 6.4 (*P* < 0.002).
**South-East Asia Region**					
India (LMIC), 4 hospitals in 1 state [41]	2007	Anaesthetists	To increase oximetry provision and perioperative monitoring	Training manual designed for Global Oximetry (GO) subproject initially used in Uganda	10 mo after training, 11 / 12 pulse oximeters were still regularly used. Anaesthetists report early detection of hypoxia, improved perioperative monitoring, and enhanced team communication.
Nepal (LMIC), 12 districts nationwide [42]	2014-2015	Anaesthesia assistants	To provide anaesthesia assistant continuing professional development	A refresher course of 5 d, 1 y with tablet-based self-learning modules and clinical case logs, regular educational mentor communication, a midcourse 2-week contact time at an anaesthesia assistant training site, regular text messaging, and clinical and MCQ examinations	Pulse oximetry was used in 98% of cases.
Thailand (UMIC), 1 hospital in Bangkok and 1 hospital in Pitsanulok [43]	Ongoing. Anaesthesia training for physicians began in 1951 and anaesthetic nurses in 1965.	Anaesthesia residents, anaesthesia fellows, and anaesthetic nurses from Thailand and nearby countries	Increasing anaesthesia workforce in the region	3-y residency for physicians, 1-y programme for nurse anaesthetists	In 2016, 25 new anaesthesiologists and 40 anaesthesia nurses trained each year. Pulse oximetry monitoring now standard.
**Western Pacific Region**					
The Philippines (LMIC): 16 hospitals in Cebu province [37]	2007	Acute care doctors and nurses	Identify pulse oximetry gaps and training needs for perioperative monitoring	1 d training course (for doctors and nurses) in the use of oximeters	Use of the oximeters throughout the project, demonstrating a change of practice.
Vietnam (LMIC): 15 hospitals in Binh Dinh province [37]	2007	Anaesthesia providers	Identify pulse oximetry gaps and training needs for perioperative monitoring	1 d Global Oximetry project workshop with refreshers 6 mo and 1 y later	All participants were able demonstrate basic oximetry use after training.

LMIC – lower middle-income country, UMIC – upper middle-income country, SSC – World Health Organization’s (WHO) Safe Surgical Checklist, d – days, mo – months, LIC – low-income country, HDU – high dependency unit, PACU – post-anaesthesia care unit, y – year, MCQ – multiple choice question

*Stratified by WHO Regions.

### Respiratory support programme expansion

Fourteen studies in six countries representing the African Region (n = 4), South-East Asia Region (n = 1), and Western Pacific Region (n = 1) described pulse oximetry training prior to oxygen programme implementation, improved oxygen use, or expansion of bubble continuous positive airway pressure (bCPAP) or ventilatory support programmes (**Table 2**). One country was low-income and five were lower-middle income. Training was focused on doctors and nurses and ranged from 45-minute lectures to 3-day workshops. Studies found increased use of ventilatory and bCPAP support after training and increased use of pulse oximetry monitoring in children with pneumonia on oxygen support. One study found decreased mortality after pulse oximetry monitoring training and oxygen introduction [55-57].

**Table 2:** Studies describing pulse oximetry training initiatives: Respiratory support programme expansion*

WHERE: country (LMIC designation), sites	WHEN: year of training	WHO: population trained	WHY: purpose of training	WHAT: structure of training	HOW: pulse oximetry related outcomes
**African Region**					
Ghana (LMIC), 1 hospital in the Sissala East District [44]	2011-2012	Medical officer and nurse anaesthetists	Introduction of non-invasive positive pressure ventilators (NIPPV)	3-d workshop	NIPPV was successfully used in 130 children and 11 adults
Kenya (LMIC), 1 hospital in Nakuru [45]	2018	Nurses and physicians	Increasing use of bCPAP for neonates	8-h course covering assessment of neonatal respiratory distress, bCPAP eligibility, and bCPAP use. Refresher training 2 and 9 mo later.	Use of bCPAP for neonates in respiratory distress increased from 2% pre-initiative to 17.6% post-initiative.
Nigeria (LMIC), 29 secondary and tertiary hospitals in Kaduna, Kano, and Niger states [46]	2017-2018	Doctors, nurses, pharmacists, and community health extension workers	Oxygen programme implementation	Train-the-trainer approach with 5-d training for trainers and 2-d training for trainees. Used curriculum based on WHO guidelines and modified from Graham et al.'s studies in Southwestern Nigeria.	Among all sites, use of pulse oximetry for pneumonia patients rose from 13.7 to 82.4% after the intervention.
Nigeria (LMIC), 12 secondary-level hospitals in 7 urban areas of Southwest Nigeria [47,48]	2014-2017	Nursing and medical staff	Implementation of oxygen system and pulse oximeters	1-h initial training session based on WHO guidelines with quarterly support visits for 18 mo	Use of pulse oximetry knowledge and practice increased after initial training but further increased and was more sustained after complete oxygen system training and implementation. Pulse oximetry introduction may have reduced the risk of death from pneumonia by ~ 50%.
Rwanda (LIC), 1 hospital in Kigali [49]	No year provided. Study was published in 2018.	Nurses, resident physicians, general practice physicians	Improving oxygen use in the emergency department	45-min training and MCQ assessment. Nurses received more specific pulse oximetry training.	Examination score increased from 60% average prior to the training to 80% average after the training. Proportion of patients with target SpO2 increased from 18.7% at baseline to 38.5% at 4 weeks and 42.0% at 12 weeks.
**South-East Asia Region**					
India (LMIC), 4 hospitals in Maharashtra [50,51]	2017-2018	Providers (unspecified)	Introducing bCPAP	Every Second Matters-Newborn and Infant Respiratory Bundle	Decreased Respiratory Severity Score on average by 1.31 with bCPAP use
**Western Pacific Region**					
Papua New Guinea (LMIC), 5 hospitals nationwide [52-54]	2004	Doctors and nurses	Determining the incidence of hypoxemia and improving hypoxemia detection in admitted children prior to oxygen programme implementation	Practical teaching and provision of protocol for pulse oximetry use	Prior to training, pulse oximetry was not used. After training, 98.9% of admitted children were evaluated with pulse oximetry. Pulse oximetry more reliably detected hypoxemia compared to clinical signs alone, which missed 29% of children with hypoxemia. Pulse oximetry was underutilised at 14-mo follow-up.
Papua New Guinea (LMIC), 39 remote health centres and district hospitals nationwide [55-57]	2016-2018	Health care workers (cadre not specified)	Implementing oxygen system	Annual training using WHO guidelines for the Clinical Use of Oxygen in Children and WHO Hospital Care for Children	Pneumonia cases and deaths, as well as referrals to escalate care, decreased after the intervention. Pulse oximetry allowed for hypoxemia monitoring and solar-powered oxygen concentrators effectively decreased morbidity and mortality in children.

LIC – low-income country, d – days, LMIC – lower middle-income country, bCPAP – bubble continuous positive airway pressure, h – hours, WHO – World Health Organization, mo – months, min – minutes, MCQ – multiple choice question

*Stratified by WHO Regions.

### Perinatal assessment and monitoring

Eight studies representing 16 countries focused on pulse oximetry training of health care workers in the perinatal period, 12 of which were in the Region of the Americas and one representing the African, Eastern Mediterranean, South-East Asia, and Western Pacific Regions (**Table 3**). Five countries were lower-middle income and 11 were upper-middle income. One study focused on perinatal monitoring of mothers, one focused on maternal and neonatal assessment and six focused on neonatal assessment and monitoring. Among those focusing on neonates, training aimed to instruct health care workers in the use of pulse oximetry to decrease the burden of retinopathy of prematurity (ROP) and as a screening tool for critical congenital heart disease (CCHD), sepsis and pneumonia. Educational efforts most often involved neonatal nurses, obstetricians and neonatologists, although one study included community health workers and another traditional birth attendants [60,64]. Commonly cited limitations in expanding oximetry included the lack of pulse oximeters, lack of trained staff, or limited staff availability. Reported study outcomes varied but many reported increased pulse oximetry knowledge or improved neonatal screening rates after training.

**Table 3:** Studies describing pulse oximetry training initiatives: perinatal assessment and monitoring*

WHERE: country (LMIC designation), sites	WHEN: year of training	WHO: population trained	WHY: purpose of training	WHAT: structure of training	HOW: pulse oximetry related outcomes
**Region of the Americas**					
Argentina (UMIC): 1 hospital in San Luis, 1 hospital in Rosario [58]	San Luis – 2019; Rosario – 2017-2018	Neonatal nurses and neonatologists	CCHD screening	SIBEN Clinical Consensus	Increased screening after training with plans for universal screening
Bolivia (LMIC): 1 hospital in La Paz, 1 hospital in Sucre [58]	2018	Staff	CCHD screening	SIBEN Clinical Consensus	Unable to routinely screen due to lack of equipment and staff
Brazil (UMIC), 5 NICUs in Rio de Janeiro [59]	2009	Neonatal nurses and nurse assistants	Improving nursing care to decrease mortality and incidence of ROP	3-mo self-administered education package	78% of qualified nurses and 82% of nurse assistants were trained and knowledge improved after training. No significant impact on survival or ROP was found.
Colombia (UMIC): 1 hospital in Barranquilla [58]	2014	Neonatal group	CCHD screening	SIBEN Clinical Consensus	Increased screening after training and screening became mandatory in 2018
Costa Rica (UMIC): Nationwide [58]	2016	Not clearly stated, screening by respiratory therapists, nurses	CCHD screening	SIBEN Clinical Consensus	Screening nationwide after training
Cuba (UMIC): not stated [58]	Cuba: not provided	Not stated, screening in some neonatal centres	CCHD screening	SIBEN Clinical Consensus	Limited screening due to pulse oximetry shortage
Dominican Republic (UMIC): Nationwide [58]	2019	Neonatologists, neonatal nurses, neonatology residents	CCHD screening	SIBEN Clinical Consensus	Screening not yet started
El Salvador (LMIC): 1 hospital in San Salvador [58]	Not provided	Not stated	CCHD screening	SIBEN Clinical Consensus	Screening not yet started
Guatemala (UMIC), Tecpán municipality [60]	Year not provided. Study published in 2018.	Traditional birth attendants	Improve detection of maternal and perinatal complications and facility referral	4-d training on perinatal complications, indications for referral, and use of the mHealth platform and device (which included a pulse oximeter)	Rate of emergency referrals to facilities increased with the mHealth intervention and training.
Honduras (LMIC): Nationwide [58]	Not provided	Staff providing neonatal care	CCHD screening	SIBEN Clinical Consensus	Increased screening after training
Paraguay (UMIC): 1 hospital in Asunción [58]	2013	Nurses	CCHD screening	SIBEN Clinical Consensus	Increased screening after training
Peru (UMIC): 1 hospital in Lima. [58]	2018	Not clearly stated, screening by nurses	CCHD screening	SIBEN Clinical Consensus	Increased screening after training, pulse oximeter provision, and hiring dedicated staff for screening
**African Region**					
South Africa (UMIC), 1 hospital in Port Elizabeth [61]	2012-2014	Paediatricians and NICU nurses	ROP prevention	Paediatric academic programme for paediatricians and educational sessions for nursing staff	More infants being screened for ROP after developing an ROP screening clinic. Training and provision of pulse oximeters and oxygen blenders made targeted oxygen supplementation possible.
**Eastern Mediterranean Region**					
Iran (LMIC), Sistan and Baluchestan provinces [62]	2014	Midwives and general practitioners	Improve management of postpartum haemorrhage	2-d training based on WHO guidelines	Pulse oximetry use increased from 0 to 81.5% after the workshop.
**South-East Asia Region**					
India (LMIC), Centres with high incidence of aggressive posterior ROP in North Karnataka [63]	2011-2015	NICU nurses and paramedical staff, paediatricians	Attempt to reduce severe ROP	ROP prevention guidelines and targeted training	More infants being are being screened for ROP but no mention of improved pulse oximetry use
**Western Pacific Region**					
China (UMIC), Rural areas [64]	2015	Public health stakeholders, clinicians, rural health workers	CCHD, pneumonia, and sepsis detection	Train the trainer model	More than 52 000 newborns have been screened
China (UMIC), Rural county hospitals in Yunnan Province [65]	2015-2016	Obstetricians and obstetric nurses	CCHD screening training	1-d hands on training	Improved knowledge via MCQs immediately after training and at 3 mo follow up 61.6% of nurses reported frequent pulse oximeter and 23.2% reported sometimes using pulse oximeters at follow up. Newborn screening rates were 90.6%-98.0%

LMIC – lower middle-income country, UMIC – upper middle-income country, CCHD – critical congenital heart defects, SIBEN – Ibero American Society of Neonatology, NICU – neonatal intensive care unit, ROP – retinopathy of prematurity, m – months, d – days, MCQ – multiple choice question

*Stratified by WHO Regions.

### Paediatric assessment and monitoring

Seven studies from four countries in the African Region, three of which were low-income countries and one was lower-middle income, focused on training health care workers caring for non-neonatal paediatric populations, especially those responsible for assessing children for pneumonia (**Table 4**). Most studies focused on training community health workers and nurses to regularly and routinely conduct vital signs assessments that included measurement of oxygen saturation. Training ranged from one-hour pulse oximetry training supplemented with additional clinical management training to a four-day emergency triaging workshop. After training, studies found increased rates of oxygen saturation measurements with several studies reporting improved morbidity and mortality among the studied population.

**Table 4:** Studies describing pulse oximetry training initiatives: paediatric assessment and monitoring*

WHERE: country (LMIC designation), sites	WHEN: year of training	WHO: population trained	WHY: purpose of training	WHAT: structure of training	HOW: pulse oximetry related outcomes
**African Region**					
Ethiopia (LIC), first-level health facilities and in the community in Sodo Zuria, Damote Sore, and Damote Gale districts [66]	2018	Health extension workers and first-level health facility workers	Assessment of children with respiratory illness	2-d iCCM/IMNCI and pulse oximetry training	83.9% of pneumonia consultations were assessed using pulse oximetry in the 2 mo following training
Malawi (LIC), Health centres in Lilongwe and Mchinji districts [67,68]	2011	Rural practitioners and community health workers	Improve identification of children with severe respiratory illness	1-d training with continued monthly mentorship visits	94.1% of children had oxygen saturation measured during the 3 y after training. Moderate agreement was found between expert oxygen saturation measurements and newly trained providers. Pulse oximetry identified fatal episodes of childhood pneumonia that did not have identified clinical signs.
Malawi (LIC), 1 hospital in Lilongwe [69]	2011	Nurses, clinicians, and vital sign assistants (VSAs)	Improve inpatient paediatric surveillance	Half-day training for nurses, 2-d training for VSAs based on PEWS concepts	Patients' vital signs, including oxygen saturation, were monitored more frequently and accurately than before the training when VSAs were included in the health care team.
Malawi (LIC), 2 clinics in the Mulanje district [70]	2019	Clinical officers and nurses	Assessing whether pulse oximeter screening in addition to IMCI education improves respiratory illness diagnosis and decreases antibiotic prescriptions in children	1-h long session using WHO pulse oximetry manual supplemented with additional recommendations	30% of children were evaluated with pulse oximeters at clinics with this capability. Clinic sites with pulse oximeters improved illness classification (diagnosed severe respiratory illnesses less frequently in children with normal oxygen saturation and more frequently in children with low oxygen saturation) and prescribed antibiotics less frequently. Pulse oximeters improved provider confidence.
Sierra Leone (LIC), 1 hospital in Freetown [71]	2009	Nurses	Improve paediatric hospital emergency medical care	4-d adapted WHO Emergency Triage Assessment and Treatment course	Decreased mortality rate immediately and 4 mo after interventions
Zambia (LMIC), 1 hospital in Lusaka [72]	2013	Nurses	Implementation of a clinical guidance tool, which included prompts for regular vital sign checks (including oxygen saturation), to improve the care of children hospitalised with pneumonia	Modified WHO recommendations for the management of acute respiratory illness were used to make the tool	Nurses believed the clinical guidance tool led to improved care through closer and more consistent monitoring and rapid identification of problems. Increase of the proportion of children with oxygen saturation ≤92% receiving oxygen on admission (83.3% vs. 93.8%) and at 48 h follow-up (76.4% vs. 95.3%)

LMIC – lower middle-income country, LIC – low-income country, d – days, iCCM/IMNCI – integrated community case management/integrated management of newborn and childhood illnesses, mo – months, PEWS – paediatric early warning score, IMCI – integrated management of childhood illness, h – hours, WHO – World Health Organization

*Stratified by WHO Regions.

### Adult assessment and monitoring

Ten studies were conducted in six countries globally, representing the Region of the Americas (n = 3), African Region (n = 2) and the Eastern Mediterranean Region (n = 1) (**Table 5**). One country was low-income, three were lower-middle income and two were upper-middle income. Studies focus on training health care workers in the assessment and monitoring of adults. Six of these studies primarily focused on improving patient monitoring and the early identification of complications by increasing the frequency of vital signs measurement. As such, these studies primarily focused their training on nurses. Three studies focused on improving trauma and emergency services in LMICs. In these studies, pulse oximetry training was not the primary focus but was included as part of comprehensive training programmes that included vital sign assessments and monitoring. Training often focused on non-physician health care workers who either triage emergency and trauma patients or provide bedside care. One study focused on training primary care providers to use pulse oximetry as an adjunct in pulmonary disease management. Training ranged from 30-minute lectures to a 7-day long workshop. Pulse oximetry use and vital sign monitoring increased after these sessions, but studies reported that additional improvements could still be made, such as further increasing oximetry use and vital sign monitoring, as well as reacting to abnormal oximetry values.

**Table 5:** Studies describing pulse oximetry training initiatives: adult assessment and monitoring

WHERE: country (LMIC designation), sites	WHEN: year of training	WHO: population trained	WHY: purpose of training	WHAT: structure of training	HOW: pulse oximetry related outcomes
**Region of the Americas**					
Ecuador (UMIC), 1 pre-hospital training site in Cuenca [73,74]	No year provided. Abstracts presented in 2017.	Pre-hospital personnel (doctors, firefighters, paramedics, ambulance operators, medical dispatchers, medical auditors, and Ministry of Health administrators)	To improve prehospital to hospital communication for injured patients	1-h long communication course and introduction of a communication checklist	Communication of pulse oximetry values increased from 37.5 to 72% after training in observed scenarios.
Haiti (LMIC), 1 hospital in Port-au-Prince [75]	2013-2014	Physicians and nurses	Implementation of an adapted WHO severe sepsis protocol and vital signs monitoring	31-h lectures on severe sepsis management, protocol implementation (including new triage and vital sign forms), and clinical data recording	After protocol implementation, 83.6% of patients had their vital signs taken a second time compared to 78.8% before the protocol implementation. Patients were more likely to have their second vital signs taken sooner after protocol implementation compared to before (140 min vs. 240 min, respectively).
Nicaragua (LMIC), Managua [76]	Year not provided. Article published in 1997.	Nurses and physicians	Critical care training, including pulse oximetry and oxyhaemoglobin dissociation curve	5-d course	No outcomes provided.
**African Region**					
South Africa (UMIC), 1 hospital in Cape Town [77]	2010	Nurses on postoperative wards	MEWS system implementation	2-h Cape Town MEWS training programme	Nurses who underwent training recorded oxygen saturation in 12.3% of patients compared to the control group, which only had 3.5% of patients with oxygen saturation recorded.
South Africa (UMIC), 6 government-sector adult hospitals in the Western Cape [78]	2014	Nurses in medical and surgical wards	MEWS system implementation	8-h MEWS and SBAR training	Nurses who underwent the training recorded pulse oximetry in 54.0% of patients compared to nurses in the control group who only recorded oxygen saturation in 17.6% of patients. Abnormal oxygen saturation values in the intervention group did not trigger assistance as they should have.
Uganda (LIC), 4 health facilities in Western Uganda [79]	2015	Health facility staff	Improved vital sign monitoring and recognition of severe illness	7 d WHO Quick Check + training programme	Pulse oximetry monitoring increased from 0.2% of patients to 19%
Uganda (LIC), 1 emergency centre in Kampala [80]	2016	Emergency centre nurses	Traumatic brain injury nursing care, vital sign monitoring, and charting	30-40-min lecture	Nurses felt that the vital signs chart improved monitoring and was useful for tracking patient progress and guiding management but felt routine vital sign monitoring may be difficult to integrate into their practice.
Uganda (LIC), Multiple regions nationwide [81]	No year provided. Abstract presented in 2019.	Primary care clinicians (doctors, clinical officers, nurses, midwives)	Improve assessment and management of pulmonary diseases in primary care, including use of pulse oximetry	iBreath	Participants report increased knowledge, skills, and improved attitudes after the course
**Eastern Mediterranean Region**					
Iran (LMIC), 1 prehospital emergency centre in Tabriz [82]	2016-2018	Prehospital emergency staff	Improve prehospital trauma care	12 session Prehospital Trauma Life Support course	Vital signs control and pulse oximetry measurement increased from 95.7 to 100% after the course

LMIC – lower middle-income country, UMIC – upper middle-income country, h – hours, WHO – World Health Organization, min – minutes, d – days, MEWS – modified early warning score, SBAR – situation, background, assessment, recommendation, LIC – low-income country.

*Stratified by WHO Regions.

## DISCUSSION

Pulse oximetry education initiatives among health care workers in LMICs focused primarily on monitoring and evaluation of patients during times of potential deterioration – such as during the perioperative period or respiratory compromise – or as a means of routine assessment or screening – such as during vital signs measurement. Training and educational programmes varied in their purpose with respect to the types of patients being targeted, the types of providers being instructed, and the depth of pulse oximetry-specific training. These initiatives included training anaesthetists or perioperative care staff, ensuring appropriate and adequate oxygen use, pulse oximetry screening for congenital diseases and infectious diseases and improving vital signs assessment for patients of all ages. Most studies focused on training doctors and nurses working in hospitals, with fewer focused on pre-hospital and community-based health care workers. Identified studies reported heterogeneous training structures for pulse oximetry, even among studies with the same goals of teaching pulse oximetry use. As such, no determination could be made about if a certain training style or method was more, or less, effective compared to another style. While these findings do not encompass all possible reasons pulse oximetry training could be conducted, they provide an overview of the training landscape as described in the literature prior to the COVID-19 pandemic.

Prior studies demonstrate that availability and use of pulse oximetry does not guarantee a proper understanding of oximetry practices, interpretation, or indications for intervention [15,83,84]. Additionally, pulse oximetry may not have been covered in primary qualification training for some health care workers [85]. Finally, knowledge without access cannot lead to improvements in patient care, just as access without proper understanding also does not lead to improvements in care. Thus, it is important for educational initiatives to occur in tandem with provision of devices, as one is not successful without the other.

In addition to addressing improved pulse oximetry availability and education, other barriers to its use also need to be assessed. Such barriers will vary based on context-specific concerns. An appropriate, thorough, and regionally specific understanding of why pulse oximetry is not utilised in particular areas where it may be beneficial should be explored, including cost, staffing shortages and power outages. This should be enabled by engagement with local health care workers to address the most relevant and pressing concerns. Future research focusing on these issues would be beneficial to inform pulse oximetry implementation and policy reforms, with a focus on local health care workers’ needs, workflows, and behaviours. Furthermore, implementation science approaches and methodologies would help standardise the way impact of such initiatives is assessed.

Globally, the COVID-19 pandemic demonstrated the need for widespread pulse oximetry availability and training for early detection of hypoxemia [9]. It laid bare the inequitable distribution of pulse oximeters and the need for increased access to pulse oximeters and health provider training in LMICs. Thousands of pulse oximeters have been distributed in LMICs throughout the pandemic by multilateral organisations and non-governmental organisations (NGOs), such as UNICEF and Lifebox [86,87]. The required rapid up scaling of pulse oximetry training and the increased availability of devices highlight a health systems area in need of strengthening. It showed that while pulse oximetry training has been conducted for many years across settings described in this review, it could benefit from additional investment. Improvements achieved since the start of the pandemic represent a momentary success, but for sustainability, widespread pulse oximetry introduction and appropriate training will need to continue [8].

This scoping review is not without limitations. This review focused on data gathered from the published literature, which we acknowledge fails to capture non-published pulse oximetry education initiatives. There may be a publication bias as to which initiatives are published and which are not. Additionally, if within a particular setting pulse oximetry is widely available and is a standard topic covered in health care schooling, educational efforts may be less likely published given its ubiquitous nature. Limitations notwithstanding, we believe this review will be beneficial to researchers, educators, and policy makers to provide a baseline understanding of many pulse oximetry education foci in LMICs prior to the COVID-19 pandemic.

## CONCLUSIONS

Pulse oximetry training initiatives have been ongoing for decades for a variety of purposes, utilising a multitude of approaches to equip various health care workers with tools to improve patient care. It is important that these initiatives continue as pulse oximetry availability and knowledge gaps remain. Neither pulse oximetry provision nor training alone is enough to bolster patient care, but sustainable solutions for both must be considered in order to meet the needs of both health care workers and patients.

## Additional material


Online Supplementary Document
