# Supplementary material for: Pulse oximetry training landscape for healthcare workers in low- and middle-income countries: A scoping review
Source: J Glob Health. 2023 Sep 22;13:04074. doi: 10.7189/jogh.13.04074 (PMC10514743; doi:10.7189/jogh.13.04074)
Supplement: Online Supplementary Document [file jogh-13-04074-s001.pdf]

Table S1. Preferred Reporting Items for Systematic reviews and Meta-Analyses extension for Scoping Reviews (PRISMA-ScR) Checklist

SECTION	ITEM	PRISMA-ScR CHECKLIST ITEM	REPORTED ON PAGE #
**TITLE**			
Title	1	Identify the report as a scoping review.	1
**ABSTRACT**			
Structured summary	2	Provide a structured summary that includes (as applicable): background, objectives, eligibility criteria, sources of evidence, charting methods, results, and conclusions that relate to the review questions and objectives.	1–2
**INTRODUCTION**			
Rationale	3	Describe the rationale for the review in the context of what is already known. Explain why the review questions/objectives lend themselves to a scoping review approach.	2–3
Objectives	4	Provide an explicit statement of the questions and objectives being addressed with reference to their key elements (e.g., population or participants, concepts, and context) or other relevant key elements used to conceptualize the review questions and/or objectives.	3
**METHODS**			
Protocol and registration	5	Indicate whether a review protocol exists; state if and where it can be accessed (e.g., a Web address); and if available, provide registration information, including the registration number.	3
Eligibility criteria	6	Specify characteristics of the sources of evidence used as eligibility criteria (e.g., years considered, language, and publication status), and provide a rationale.	4
Information sources*	7	Describe all information sources in the search (e.g., databases with dates of coverage and contact with authors to identify additional sources), as well as the date the most recent search was executed.	3–4
Search	8	Present the full electronic search strategy for at least 1 database, including any limits used, such that it could be repeated.	Supplement 2
Selection of sources of evidence†	9	State the process for selecting sources of evidence (i.e., screening and eligibility) included in the scoping review.	4
Data charting process‡	10	Describe the methods of charting data from the included sources of evidence (e.g., calibrated forms or forms that have been tested by the team before their use, and whether data charting was done independently or in duplicate) and any processes for obtaining and confirming data from investigators.	4–5
Data items	11	List and define all variables for which data were sought and any assumptions and simplifications made.	4–5
Critical appraisal of individual sources of evidence§	12	If done, provide a rationale for conducting a critical appraisal of included sources of evidence; describe the methods used and how this information was used in any data synthesis (if appropriate).	n/a
Synthesis of results	13	Describe the methods of handling and summarizing the data that were charted.	4–5

SECTION	ITEM	PRISMA-ScR CHECKLIST ITEM	REPORTED ON PAGE #
**RESULTS**			
Selection of sources of evidence	14	Give numbers of sources of evidence screened, assessed for eligibility, and included in the review, with reasons for exclusions at each stage, ideally using a flow diagram.	5, Figure 1
Characteristics of sources of evidence	15	For each source of evidence, present characteristics for which data were charted and provide the citations.	Tables 1–5
Critical appraisal within sources of evidence	16	If done, present data on critical appraisal of included sources of evidence (see item 12).	n/a
Results of individual sources of evidence	17	For each included source of evidence, present the relevant data that were charted that relate to the review questions and objectives.	Tables 1–5
Synthesis of results	18	Summarize and/or present the charting results as they relate to the review questions and objectives.	5–8
**DISCUSSION**			
Summary of evidence	19	Summarize the main results (including an overview of concepts, themes, and types of evidence available), link to the review questions and objectives, and consider the relevance to key groups.	8–10
Limitations	20	Discuss the limitations of the scoping review process.	10
Conclusions	21	Provide a general interpretation of the results with respect to the review questions and objectives, as well as potential implications and/or next steps.	10
**FUNDING**			
Funding	22	Describe sources of funding for the included sources of evidence, as well as sources of funding for the scoping review. Describe the role of the funders of the scoping review.	10

JBI = Joanna Briggs Institute; PRISMA-ScR = Preferred Reporting Items for Systematic reviews and Meta-Analyses extension for Scoping Reviews.
* Where *sources of evidence* (see second footnote) are compiled from, such as bibliographic databases, social media platforms, and Web sites.
† A more inclusive/heterogeneous term used to account for the different types of evidence or data sources (e.g., quantitative and/or qualitative research, expert opinion, and policy documents) that may be eligible in a scoping review as opposed to only studies. This is not to be confused with *information sources* (see first footnote).
‡ The frameworks by Arksey and O'Malley (6) and Levac and colleagues (7) and the JBI guidance (4, 5) refer to the process of data extraction in a scoping review as data charting.
§ The process of systematically examining research evidence to assess its validity, results, and relevance before using it to inform a decision. This term is used for items 12 and 19 instead of "risk of bias" (which is more applicable to systematic reviews of interventions) to include and acknowledge the various sources of evidence that may be used in a scoping review (e.g., quantitative and/or qualitative research, expert opinion, and policy document).

From: Tricco AC, Lillie E, Zarin W, O'Brien KK, Colquhoun H, Levac D, et al. PRISMA Extension for Scoping Reviews (PRISMAScR): Checklist and Explanation. Ann Intern Med. 2018;169:467–473. doi: 10.7326/M18-0850.

Table S2. PubMed search terms

History and Search Details

Download

Delete

Search	Actions	Details	Query	Results	Time
#16			Search: **#10 AND #15**	219	18:56:06
#15			Search: **"education"[Subheading] or education[mesh] or train*[tw] or educat*[tw] or workshop[tw] or teach*[tw] or precedure*[tw] or evaluat*[tw] or implement*[tw]**	5,520,140	18:54:49
#10			Search: **#8 AND #9**	218	12:04:37
#8			Search: **#6 NOT #7**	1,605	12:01:29
#7			Search: **(animals[mh] NOT humans[mh])**	4,718,526	12:01:12
#6			Search: **#1 AND #5**	1,670	12:00:45
#5			Search: **#3 or #4**	2,052,788	12:00:29
#4			Search: **afghanistan[MeSH] OR albania[MeSH] OR algeria[MeSH] OR american samoa[MeSH] OR angola[MeSH] OR antigua and barbuda[MeSH] OR argentina[MeSH] OR armenia[MeSH] OR aruba[MeSH] OR azerbaijan[MeSH] OR bahrain[MeSH] OR bangladesh[MeSH] OR barbados[MeSH] OR republic of belarus[MeSH] OR belize[MeSH] OR**	1,097,942	11:59:52

Search	Actions	Details	Query	Results	Time
			benin[MeSH] OR bhutan[MeSH] OR bolivia[MeSH] OR bosnia and herzegovina[MeSH] OR botswana[MeSH] OR brazil[MeSH] OR bulgaria[MeSH] OR burkina faso[MeSH] OR burundi[MeSH] OR cabo verde[MeSH] OR cambodia[MeSH] OR cameroon[MeSH] OR central african republic[MeSH] OR chad[MeSH] OR chile[MeSH] OR china[MeSH] OR colombia[MeSH] OR comoros[MeSH] OR democratic republic of the congo[MeSH] OR congo[MeSH] OR costa rica[MeSH] OR cote d'ivoire[MeSH] OR croatia[MeSH] OR cuba[MeSH] OR cyprus[MeSH] OR czech republic[MeSH] OR djibouti[MeSH] OR dominica[MeSH] OR dominican republic[MeSH] OR ecuador[MeSH] OR egypt[MeSH] OR el salvador[MeSH] OR equatorial guinea[MeSH] OR eritrea[MeSH] OR estonia[MeSH] OR swaziland[MeSH] OR ethiopia[MeSH] OR fiji[MeSH] OR gabon[MeSH] OR gambia[MeSH] OR georgia (republic)[MeSH] OR ghana[MeSH] OR gibraltar[MeSH] OR greece[MeSH] OR grenada[MeSH] OR guam[MeSH] OR guatemala[MeSH] OR guinea[MeSH] OR guinea bissau[MeSH] OR guyana[MeSH] OR haiti[MeSH] OR honduras[MeSH] OR hungary[MeSH] OR india[MeSH] OR indonesia[MeSH] OR iran[MeSH] OR iraq[MeSH] OR jamaica[MeSH] OR jordan[MeSH] OR kazakhstan[MeSH] OR kenya[MeSH] OR democratic people's republic of korea[MeSH] OR republic of korea[MeSH] OR kosovo[MeSH] OR kyrgyzstan[MeSH] OR laos[MeSH] OR latvia[MeSH] OR		

Search	Actions	Details	Query	Results	Time
			lebanon[MeSH] OR lesotho[MeSH] OR liberia[MeSH] OR libya[MeSH] OR lithuania[MeSH] OR macau[MeSH] OR macedonia (republic)[MeSH] OR madagascar[MeSH] OR malawi[MeSH] OR malaysia[MeSH] OR indian ocean islands[MeSH] OR mali[MeSH] OR malta[MeSH] OR micronesia[MeSH] OR palau[MeSH] OR mauritania[MeSH] OR mauritius[MeSH] OR mexico[MeSH] OR moldova[MeSH] OR mongolia[MeSH] OR montenegro[MeSH] OR morocco[MeSH] OR mozambique[MeSH] OR myanmar[MeSH] OR namibia[MeSH] OR nepal[MeSH] OR netherlands antilles[MeSH] OR nicaragua[MeSH] OR niger[MeSH] OR nigeria[MeSH] OR oman[MeSH] OR pakistan[MeSH] OR panama[MeSH] OR papua new guinea[MeSH] OR paraguay[MeSH] OR peru[MeSH] OR philippines[MeSH] OR poland[MeSH] OR portugal[MeSH] OR puerto rico[MeSH] OR romania[MeSH] OR russia[MeSH] OR rwanda[MeSH] OR samoa[MeSH] OR sao tome and principe[MeSH] OR saudi arabia[MeSH] OR senegal[MeSH] OR serbia[MeSH] OR seychelles[MeSH] OR sierra leone[MeSH] OR slovakia[MeSH] OR slovenia[MeSH] OR melanesia[MeSH] OR somalia[MeSH] OR south africa[MeSH] OR south sudan[MeSH] OR sri lanka[MeSH] OR saint kitts and nevis[MeSH] OR saint lucia[MeSH] OR saint vincent and the grenadines[MeSH] OR sudan[MeSH] OR suriname[MeSH] OR syria[MeSH] OR tajikistan[MeSH] OR		

Search	Actions	Details	Query	Results	Time
			**tanzania[MeSH] OR thailand[MeSH] OR timor leste[MeSH] OR togo[MeSH] OR tonga[MeSH] OR trinidad and tobago[MeSH] OR tunisia[MeSH] OR turkey (republic)[MeSH] OR turkmenistan[MeSH] OR uganda[MeSH] OR ukraine[MeSH] OR uruguay[MeSH] OR uzbekistan[MeSH] OR vanuatu[MeSH] OR venezuela[MeSH] OR vietnam[MeSH] OR middle east[MeSH] OR yemen[MeSH] OR yugoslavia[MeSH] OR zambia[MeSH] OR zimbabwe[MeSH] OR africa south of the sahara[MeSH] OR africa, central[MeSH] OR africa, northern[MeSH] OR africa, southern[MeSH] OR africa, eastern[MeSH] OR africa, western[MeSH] OR west indies[MeSH] OR indian ocean islands[MeSH] OR caribbean region[MeSH] OR central america[MeSH] OR latin america[MeSH] OR south america[MeSH] OR asia, central[MeSH] OR asia, northern[MeSH] OR asia, southeastern[MeSH] OR asia, western[MeSH] OR europe, eastern[MeSH] OR developing countries[MeSH]**		
#3			Search: **afghanistan[Text Word] OR albania[Text Word] OR algeria[Text Word] OR american samoa[Text Word] OR angola[Text Word] OR antigua[Text Word] OR barbuda[Text Word] OR argentina[Text Word] OR armenia[Text Word] OR armenian[Text Word] OR aruba[Text Word] OR azerbaijan[Text Word] OR bahrain[Text**	1,987,802	11:59:40

Search	Actions	Details	Query	Results	Time
			Word] OR bangladesh[Text Word] OR barbados[Text Word] OR belarus[Text Word] OR byelarus[Text Word] OR belorussia[Text Word] OR byelorussian[Text Word] OR belize[Text Word] OR british honduras[Text Word] OR benin[Text Word] OR dahomey[Text Word] OR bhutan[Text Word] OR bolivia[Text Word] OR bosnia[Text Word] OR herzegovina[Text Word] OR botswana[Text Word] OR bechuanaland[Text Word] OR brazil[Text Word] OR brasil[Text Word] OR bulgaria[Text Word] OR burkina faso[Text Word] OR burkina fasso[Text Word] OR upper volta[Text Word] OR burundi[Text Word] OR urundi[Text Word] OR cabo verde[Text Word] OR cape verde[Text Word] OR cambodia[Text Word] OR kampuchea[Text Word] OR khmer republic[Text Word] OR cameroon[Text Word] OR cameron[Text Word] OR cameroun[Text Word] OR central african republic[Text Word] OR ubangi shari[Text Word] OR chad[Text Word] OR chile[Text Word] OR china[Text Word] OR colombia[Text Word] OR comoros[Text Word] OR comoro islands[Text Word] OR mayotte[Text Word] OR congo[Text Word] OR zaire[Text Word] OR costa rica[Text Word] OR cote d'ivoire[Text Word] OR cote d'ivoire[Text Word] OR cote d'ivoire[Text Word] OR ivory coast[Text Word] OR croatia[Text Word] OR cuba[Text Word] OR cyprus[Text Word] OR czech republic[Text Word] OR		

Search	Actions	Details	Query	Results	Time
			czechoslovakia[Text Word] OR djibouti[Text Word] OR french somaliland[Text Word] OR dominica[Text Word] OR dominican republic[Text Word] OR ecuador[Text Word] OR egypt[Text Word] OR united arab republic[Text Word] OR el salvador[Text Word] OR equatorial guinea[Text Word] OR spanish guinea[Text Word] OR eritrea[Text Word] OR estonia[Text Word] OR eswatini[Text Word] OR swaziland[Text Word] OR ethiopia[Text Word] OR fiji[Text Word] OR gabon[Text Word] OR gabonese republic[Text Word] OR gambia[Text Word] OR georgia[Text Word] OR georgian[Text Word] OR ghana[Text Word] OR gold coast[Text Word] OR gibraltar[Text Word] OR greece[Text Word] OR grenada[Text Word] OR guam[Text Word] OR guatemala[Text Word] OR guinea[Text Word] OR guyana[Text Word] OR guiana[Text Word] OR haiti[Text Word] OR hispaniola[Text Word] OR honduras[Text Word] OR hungary[Text Word] OR india[Text Word] OR indonesia[Text Word] OR timor[Text Word] OR iran[Text Word] OR iraq[Text Word] OR isle of man[Text Word] OR jamaica[Text Word] OR jordan[Text Word] OR kazakhstan[Text Word] OR kazakh[Text Word] OR kenya[Text Word] OR korea[Text Word] OR kosovo[Text Word] OR kyrgyzstan[Text Word] OR kirghizia[Text Word] OR kirgizstan[Text Word] OR kyrgyz republic[Text Word] OR kirghiz[Text Word] OR laos[Text Word] OR lao		

Search	Actions	Details	Query	Results	Time
			pdr[Text Word] OR lao people's democratic republic[Text Word] OR latvia[Text Word] OR lebanon[Text Word] OR lesotho[Text Word] OR basutoland[Text Word] OR liberia[Text Word] OR libya[Text Word] OR libyan arab jamahiriya[Text Word] OR lithuania[Text Word] OR macau[Text Word] OR macao[Text Word] OR macedonia[Text Word] OR madagascar[Text Word] OR malagasy republic[Text Word] OR malawi[Text Word] OR nyasaland[Text Word] OR malaysia[Text Word] OR maldives[Text Word] OR indian ocean[Text Word] OR mali[Text Word] OR malta[Text Word] OR micronesia[Text Word] OR kiribati[Text Word] OR marshall islands[Text Word] OR nauru[Text Word] OR northern mariana islands[Text Word] OR palau[Text Word] OR tuvalu[Text Word] OR mauritania[Text Word] OR mauritius[Text Word] OR mexico[Text Word] OR moldova[Text Word] OR moldovian[Text Word] OR mongolia[Text Word] OR montenegro[Text Word] OR morocco[Text Word] OR ifni[Text Word] OR mozambique[Text Word] OR portuguese east africa[Text Word] OR myanmar[Text Word] OR burma[Text Word] OR namibia[Text Word] OR nepal[Text Word] OR netherlands antilles[Text Word] OR nicaragua[Text Word] OR niger[Text Word] OR nigeria[Text Word] OR oman[Text Word] OR muscat[Text Word] OR pakistan[Text Word] OR panama[Text Word] OR papua new guinea[Text Word] OR paraguay[Text Word] OR		

Search	Actions	Details	Query	Results	Time
			peru[Text Word] OR philippines[Text Word] OR philipines[Text Word] OR phillipines[Text Word] OR phillippines[Text Word] OR poland[Text Word] OR polish people's republic[Text Word] OR portugal[Text Word] OR portuguese republic[Text Word] OR puerto rico[Text Word] OR romania[Text Word] OR russia[Text Word] OR russian federation[Text Word] OR ussr[Text Word] OR soviet union[Text Word] OR union of soviet socialist republics[Text Word] OR rwanda[Text Word] OR ruanda[Text Word] OR samoa[Text Word] OR pacific islands[Text Word] OR polynesia[Text Word] OR samoan islands[Text Word] OR sao tome and principe[Text Word] OR saudi arabia[Text Word] OR senegal[Text Word] OR serbia[Text Word] OR seychelles[Text Word] OR sierra leone[Text Word] OR slovakia[Text Word] OR slovak republic[Text Word] OR slovenia[Text Word] OR melanesia[Text Word] OR solomon island[Text Word] OR solomon islands[Text Word] OR norfolk island[Text Word] OR somalia[Text Word] OR south africa[Text Word] OR south sudan[Text Word] OR sri lanka[Text Word] OR ceylon[Text Word] OR saint kitts and nevis[Text Word] OR st kitts and nevis[Text Word] OR saint lucia[Text Word] OR st lucia[Text Word] OR saint vincent[Text Word] OR st vincent[Text Word] OR grenadines[Text Word] OR sudan[Text Word] OR suriname[Text Word] OR surinam[Text Word] OR syria[Text Word] OR syrian arab republic[Text		

Search	Actions	Details	Query	Results	Time
			Word] OR tajikistan[Text Word] OR tadjikistan[Text Word] OR tadzhikistan[Text Word] OR tadzhik[Text Word] OR tanzania[Text Word] OR tanganyika[Text Word] OR thailand[Text Word] OR siam[Text Word] OR timor leste[Text Word] OR east timor[Text Word] OR togo[Text Word] OR togolese republic[Text Word] OR tonga[Text Word] OR trinidad[Text Word] OR tobago[Text Word] OR tunisia[Text Word] OR turkey[Text Word] OR turkmenistan[Text Word] OR turkmen[Text Word] OR uganda[Text Word] OR ukraine[Text Word] OR uruguay[Text Word] OR uzbekistan[Text Word] OR uzbek[Text Word] OR vanuatu[Text Word] OR new hebrides[Text Word] OR venezuela[Text Word] OR vietnam[Text Word] OR viet nam[Text Word] OR middle east[Text Word] OR west bank[Text Word] OR gaza[Text Word] OR palestine[Text Word] OR yemen[Text Word] OR yugoslavia[Text Word] OR zambia[Text Word] OR zimbabwe[Text Word] OR northern rhodesia[Text Word] OR global south[Text Word] OR africa south of the sahara[Text Word] OR sub saharan africa[Text Word] OR subsaharan africa[Text Word] OR central africa[Text Word] OR north africa[Text Word] OR northern africa[Text Word] OR magreb[Text Word] OR maghrib[Text Word] OR sahara[Text Word] OR southern africa[Text Word] OR east africa[Text Word] OR eastern africa[Text Word] OR west africa[Text Word] OR western africa[Text Word]		

Search	Actions	Details	Query	Results	Time
			OR west indies[Text Word] OR indian ocean islands[Text Word] OR caribbean[Text Word] OR central america[Text Word] OR latin america[Text Word] OR south america[Text Word] OR central asia[Text Word] OR north asia[Text Word] OR northern asia[Text Word] OR southeastern asia[Text Word] OR south eastern asia[Text Word] OR southeast asia[Text Word] OR south east asia[Text Word] OR western asia[Text Word] OR east europe[Text Word] OR eastern europe[Text Word] OR developing country[Text Word] OR developing countries[Text Word] OR developing nation[Text Word] OR developing nations[Text Word] OR developing population[Text Word] OR developing populations[Text Word] OR developing world[Text Word] OR less developed country[Text Word] OR less developed countries[Text Word] OR less developed nation[Text Word] OR less developed nations[Text Word] OR less developed world[Text Word] OR lesser developed countries[Text Word] OR lesser developed nations[Text Word] OR under developed country[Text Word] OR under developed countries[Text Word] OR under developed nations[Text Word] OR under developed world[Text Word] OR underdeveloped country[Text Word] OR underdeveloped countries[Text Word] OR underdeveloped nation[Text Word] OR underdeveloped nations[Text Word] OR underdeveloped population[Text Word] OR underdeveloped populations[Text		

Search	Actions	Details	Query	Results	Time
			Word] OR underdeveloped world[Text Word] OR middle income country[Text Word] OR middle income countries[Text Word] OR middle income nation[Text Word] OR middle income nations[Text Word] OR middle income population[Text Word] OR middle income populations[Text Word] OR low income country[Text Word] OR low income countries[Text Word] OR low income nation[Text Word] OR low income nations[Text Word] OR low income population[Text Word] OR low income populations[Text Word] OR lower income country[Text Word] OR lower income countries[Text Word] OR lower income nations[Text Word] OR lower income population[Text Word] OR lower income populations[Text Word] OR underserved countries[Text Word] OR underserved nations[Text Word] OR underserved population[Text Word] OR underserved populations[Text Word] OR under served population[Text Word] OR under served populations[Text Word] OR deprived countries[Text Word] OR deprived population[Text Word] OR deprived populations[Text Word] OR poor country[Text Word] OR poor countries[Text Word] OR poor nation[Text Word] OR poor nations[Text Word] OR poor population[Text Word] OR poor populations[Text Word] OR poor world[Text Word] OR poorer countries[Text Word] OR poorer nations[Text Word] OR poorer population[Text Word] OR poorer populations[Text Word] OR		

Search	Actions	Details	Query	Results	Time
			**developing economy[Text Word] OR developing economies[Text Word] OR less developed economy[Text Word] OR less developed economies[Text Word] OR underdeveloped economies[Text Word] OR middle income economy[Text Word] OR middle income economies[Text Word] OR low income economy[Text Word] OR low income economies[Text Word] OR lower income economies[Text Word] OR low gdp[Text Word] OR low gnp[Text Word] OR low gross domestic[Text Word] OR low gross national[Text Word] OR lower gdp[Text Word] OR lower gross domestic[Text Word] OR lmic[Text Word] OR lmics[Text Word] OR third world[Text Word] OR lami country[Text Word] OR lami countries[Text Word] OR transitional country[Text Word] OR transitional countries[Text Word] OR emerging economies[Text Word] OR emerging nation[Text Word] OR emerging nations[Text Word] or human development index[tw] or low development index[tw] or middle development index[tw]**		
#1			Search: **((pulse[Text Word]) AND (oximetry[MeSH Terms] or oximetr*[tw] or oximeter*[tw]) or "Transcutaneous Blood Gas Monitor*"[tw] or "blood oxygen measure*"[tw] or "oxygen saturation"[tw] or lifebox[tw])**	31,914	11:39:17
